# Genomic characterization of *Mycoplasma bovis, Mannheimia haemolytica*, and bovine viral diarrhea virus 1 from a bovine respiratory disease complex case

**DOI:** 10.1186/s12863-026-01456-0

**Published:** 2026-07-11

**Authors:** Daniel W. Nielsen, David J. Holthausen, Anna K. Goldkamp, Darrell O. Bayles, Beate M. Crossley, Harish Menghwar, Eduardo Casas, Rohana P. Dassanayake

**Affiliations:** 1 Ruminant Diseases and Immunology Research Unit, National Animal Disease Center, USDA, Agricultural Research Service, Ames, IA 50010 USA; 2 Infectious Bacterial Diseases Research Unit, National Animal Disease Center, USDA, Agricultural Research Service, Ames, IA 50010 USA; 3 California Animal Health and Food Safety Laboratory, Medicine & Epidemiology, University of California, Davis, Davis, CA, 95616 USA; 4 ARS Research Participation Program, Oak Ridge Institute for Science and Education (ORISE), Oak Ridge, TN, 37831 USA

**Keywords:** *Mycoplasma bovis*, *Mannheimia haemolytica*, Bovine viral diarrhea virus (BVDV), Bovine respiratory disease (BRD)

## Abstract

**Background:**

Bovine respiratory disease (BRD) complex is a result of the interactions among pathogens, environmental factors, host factors, and management practices. Bacterial pathogens that contribute to BRD include *Mycoplasma* (*Mycoplasmopsis*) *bovis*, *Mannheimia haemolytica*, *Pasteurella multocida*, and *Histophilus somni*. Viral agents that contribute to BRD include bovine herpesvirus 1, bovine viral diarrhea virus (BVDV), bovine respiratory syncytial virus, parainfluenza-3 virus, bovine coronavirus, and influenza D virus.

**Results:**

A case of BRD was submitted to the California Animal Health and Food Safety Laboratory for diagnostic evaluation. Bacterial and viral pathogens were isolated from a pneumonic lung sample including *M. bovis*, *M. haemolytica*, and BVDV. The DNAs/RNA of the pathogens were extracted, sequenced, analyzed, and compared to publicly available sequencing data. The genome of *M. bovis* was identified as a unique sequence type (ST299) with two novel alleles (*gltX* 28 and *gpsA* 32) and contained unique variable surface lipoproteins. However, the *M. bovis* genome also phylogenetically clustered with the *M. bovis* type strain PG45. The genome of *M. haemolytica* was similar to other publicly available *M. haemolytica* genomes, as it was assigned to sequence type 1 (ST1) and serotype 1. Finally, the genome of the isolated BVDV strain showed high similarity to the Singer (BVDV-1a) vaccine strain.

**Conclusion:**

As BRD is a complex, case studies in which the pathogens co-occur in the lung are important to report. Here, we report the complete genomes of three pathogens isolated from a single pneumonic lung sample. Notably, the *M. bovis* genome represents a unique sequence type. Future work should investigate the influence of the nine amino acid changes between the UCD-16 BVDV isolate and the Singer strain on disease pathogenesis.

**Supplementary Information:**

The online version contains supplementary material available at https://doi.org/10.1186/s12863-026-01456-0.

## Background

Bovine respiratory disease (BRD) complex, also known as shipping fever, is a multifactorial complex involving a combination of pathogens, host factors, environmental factors, and management practices. BRD is characterized by the manifestation of pneumonia as a result of stressors, such as transport, overcrowding, and poor ventilation, which compromises the host immune system and increases susceptibility to pathogens [1]. BRD is a frequently occurring and costly disease that affects the North American beef industry. Infectious agents associated with BRD include bacterial and viral pathogens. Bacterial pathogens are often the secondary agents of clinical disease, with viral infections usually being pre-existing or concurrent with bacterial infection [2]. The BRD implicated bacterial pathogens include *Mycoplasma bovis* (*Mycoplasmopsis bovis*) and members of the *Pasteurellaceae* family (*Mannheimia haemolytica*, *Pasteurella multocida*, and *Histophilus somni*) [3]. Bovine respiratory disease associated viral pathogens include bovine herpesvirus 1, bovine viral diarrhea virus (BVDV), bovine respiratory syncytial virus, parainfluenza-3 virus, bovine coronaviruses, and influenza D virus [3].

Co-infection and secondary infections play a significant role in the development of clinical pneumonia associated with the BRD complex. Previous studies have found an association between bacterial genera in the BRD complex, such as *Mycoplasma* and members of the family *Pasteurellaceae* [4, 5]. The nasopharyngeal microbiota of feedlot cattle include a greater abundance of *Mycoplasma* and *Mannheimia* 60 days post feedlot-entry compared to when they entered the feedlot [6]. In a 16S rRNA study, *Mycoplasma* and *Pasteurellaceae* were the most abundant operational taxonomic units (OTUs) identified in the lungs and lymph nodes of calves that died from BRD [5]. *Mycoplasma* also had greater relative abundances in post-mortem lung samples collected from fatal cases of BRD in dairy calves compared to clinically healthy calves without lung lesions [5]. In cattle, *M. bovis* causes respiratory disease, otitis media, arthritis, reproductive disorders, and mastitis [7], and different epidemiological schemes have been developed to identify *M. bovis* strains or sequence types [8–10].

Bovine viral diarrhea virus (BVDV) is a virus of significant prevalence and economic concern for the cattle industry in the United States and across the world [11, 12]. BVDV is a positive-sense single-stranded RNA *Pestivirus* of the family *Flaviviridae* [13]. There are two known genotypes of BVDV (BVDV1 and BVDV2), currently classified as *Pestivirus bovis* and *Pestivirus tauri*, which are divided into 25 subgenotypes (BVDV1a-u and BVDV2a-d) [14]. Although vaccines against BVDV have been developed and have been available since the 1960s, persistently infected calf reservoirs from transplacental infection have hindered control efforts and contributed to the evolution of viruses with high genetic diversity [15–17]. BVDV is divided into two biotypes, cytopathic (CP), and non-cytopathic (NCP), based upon whether cytopathic effect develops during cell culture infection [18]. Only NCP BVDV strains establish persistent infections [19]. Beyond singular infections that are most notably associated with reproductive losses, BVDV contributes to BRD co-infections [20, 21].

Here, we isolated viral and bacterial pathogens from a case of BRD, performed whole genome sequencing, and compared the genomes of the isolated pathogens with publicly available genome data. The in-depth characterization of this case provides important information on the co-occurrence of the BRD pathogens, as it was not limited to either viral or bacterial species-specific surveillance.

## Results

A pneumonic lung tissue sample was received at the California Animal Health and Food Safety Laboratory at the University of California, Davis on October 30, 2020, from a 500-pound bovine that was co-mingled at a multi-source location in Merced County, California. The sample was transferred to the USDA Ruminant Diseases and Immunology Research Unit’s BVDV laboratory based upon the initial diagnostic testing positive for BVDV. A proprietary qPCR-based bovine respiratory molecular diagnostic panel was completed by the Iowa State University Veterinary Diagnostic Laboratory upon receipt of the sample per their standard operating procedures. The sample tested positive for BVDV, *M. bovis*, *M. haemolytica*, and *H. somni* (Table 1). As *H. somni* could not be recovered by culture, the genomic analysis was performed only on *M. bovis*, *M. haemolytica*, and BVDV.

**Table 1:** qPCR-based BRD diagnostic panel results

Animal ID	Specimen	Target Agents	Ct/Result
D2012926	Swab	Bovine herpesvirus 1	≥35/Negative
		Bovine coronavirus	≥35/Negative
		**Bovine viral diarrhea virus**	**28.3/Positive**
		Bovine respiratory syncytial virus	≥35/Negative
		*Pasteurella multocida*	≥35/Negative
		*Mycoplasma bovis*	**26.5/Positive**
		*Mannheimia haemolytica*	**22.9/Positive**
		*Histophilus somni*	**30.2/Positive**

### Genomic comparisons of M. bovis isolate BC10 with publicly available M. bovis

**Fig. 1:**
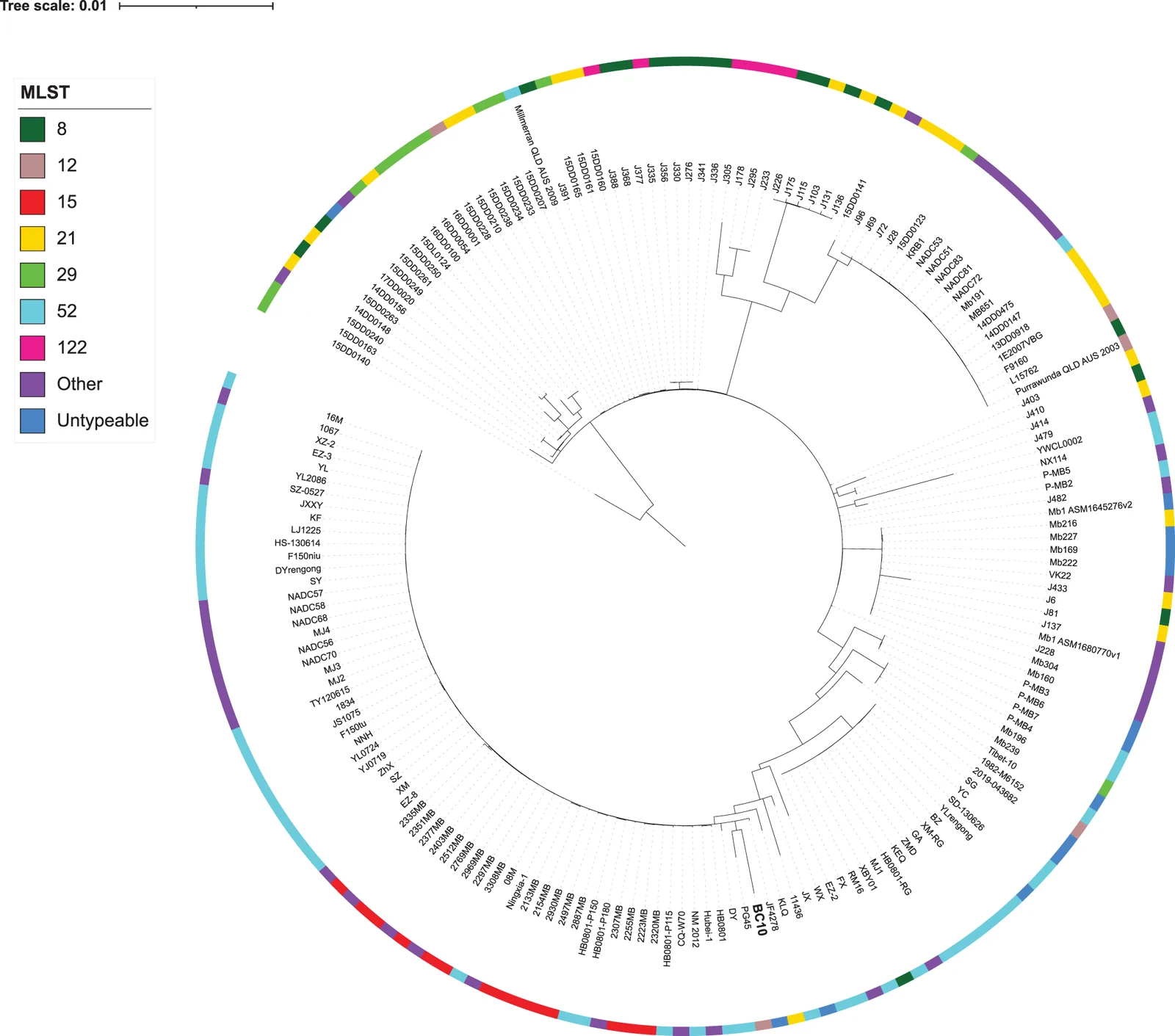
Maximum likelihood phylogenetic tree of the core genome of BC10 with publicly available *M. bovis* strains with non-redundant core genomes (*n* = 179). BC10 shared a most common recent ancestor with PG45

The *M. bovis* sample was named BC10, and it was compared to publicly available genomes. ST15 (17/183), ST21 (23/183), and ST52 (51/183) were the most common sequence types of the dataset (Supplemental File 1). Overall, the multilocus sequence typing (MLST) was not representative of the core genome phylogenetic tree for *M. bovis* (Fig. 1). Along with twelve other *M. bovis* strains, BC10 was found to be unassignable by the Register et al. MLST scheme (Fig. 1) [8]. Further analysis revealed that BC10 contained two novel alleles (*gltX* 28 and *gpsA* 32) among the seven genes used in the MLST typing. With the novel alleles, BC10 was identified as a novel ST, ST299. When the genome of BC10 was analyzed, BC10 was found to share a most common recent ancestor with PG45. PG45, also known as Donetta, is the *M. bovis* type strain that was isolated in 1961 from a mastitis outbreak [22, 23]. Compared to *M. agalactiae* strain PG2, PG45 has an inversion of 483-kb [24]. BC10 has an inversion of 253-kb compared to PG2, and 613-kb compared to PG45, demonstrating similarity to *M. bovis* strain HB0801 when compared to PG45 [25]. Although PG45 was assigned to ST12 (Fig. 1), the shared most common recent ancestor suggests that PG45 continues to be relatively representative of some circulating *M. bovis* strains.

After analyzing the core genome, the presence of *M. bovis* virulence-associated genes was queried [26]. All genes but *mbfN*, encoding *M. bovis* fibronectin-binding lipoprotein, were present in BC10; *mbfN* was also absent in the majority of *M. bovis* genomes (Table 2). When the pangenome was examined, BC10 did not carry any unique proteins when compared to all other *M. bovis* genomes. However, the pangenome analysis did identify that BC10 had a variable surface protein that differed from other *M. bovis* strains with a 97.2% nucleic acid identity. Highly variable surface lipoproteins (encoded by *vsp* genes) are known to contribute to the rapid evolution of *M. bovis* and immune evasion [27]. PG45 carries 13 different single-copy *vsp* genes in a 17.8-kb region with a genome-wide total of 26 *vsp* genes [28]. The BC10 genome contained a total of 30 *vsp* genes, with 16 *vsp* genes carried in a 20.7-kb region. When translated in silico, none of the variable surface lipoproteins between the BC10 and PG45 genomes shared an amino acid sequence with 100% identity. No antimicrobial resistance genes were found using ResFinder.

**Table 2:** Presence of virulence-associated genes with greater than 90% coverage and identity in the BC10 and GenBank collection genomes

Queried Nucleotide Sequence	BC10	GenBank Collection (n = 183)
*eno*	+	183 (100%)
*fba*	+	183 (100%)
*lppA*	+	134 (73.2%)
*mbfN*	-	9 (4.9%)
Mbov_503	+	183 (100%)
MBOV_RS03440	+	183 (100%)
*milA*	+	145 (79.2%)
*nox*	+	183 (100%)
*trmFO*	+	174 (95.1%)
*vpmax*	+	81 (44.3%)

### *Genomic comparisons of M. haemolytica* isolate *BC2 with publicly available M. haemolytica*

The *M. haemolytica* sample was named BC2, and it was compared to publicly available genomes. BC2 was assigned to genotype 2 and ST1, the most common sequence type of the *M. haemolytica* genomes (168/382). The core genome phylogenetic tree indicated that ST1 and serotype 1 assignments were both broadly distributed across the phylogenetic tree (Figure S1). When the pangenome was examined, BC2 did not carry any unique genes when compared to all other *M. haemolytica* genomes. No antimicrobial resistance genes were observed when screened with ResFinder. BC2 had a virulence-associated gene profile comparable to other serotype 1 genomes (Figure S2).

### Genomic comparison of UCD-16 with known bovine viral diarrhea virus strains

**Fig. 2:**
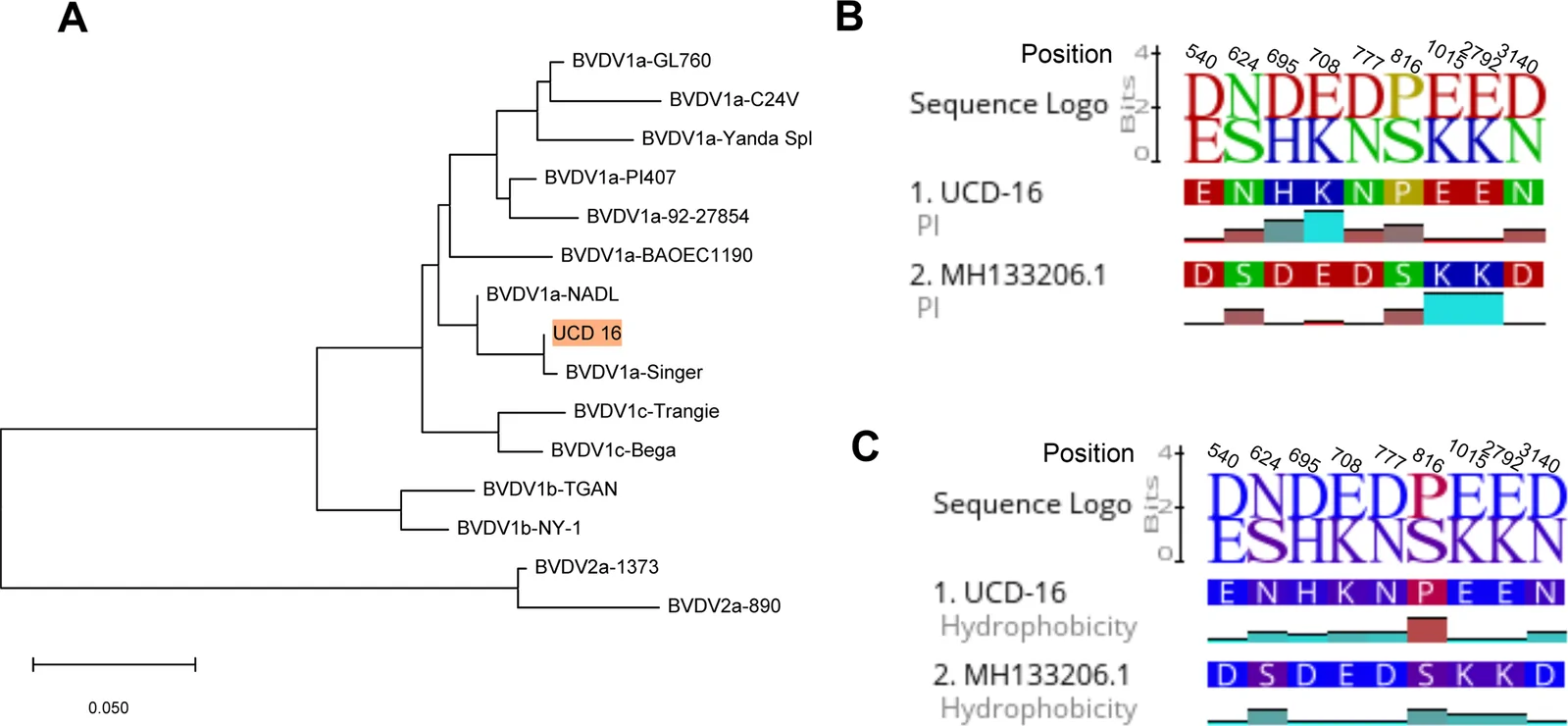
(**A**) Phylogenetic dendrogram of UCD-16 compared with a selection of known BVDV1a, BVDV1b, BVDV1c, and BVDV2a strains’ 5` UTR region generated via ClustalW alignment and a maximum likelihood tree using MEGA12 software. Branch supports were estimated via 1,000 bootstrap replicates and Poisson correction to calculate evolutionary distances. UCD-16 is highlighted in orange. (**B,C**) Whole genome amino acid changes between UCD-16 and BVDV1a-Singer (MH133206.1) demonstrating differences in isoelectric point (PI) (**B**) and hydrophobicity (**C**) of residues

The BVDV isolate from the sample, named UCD-16, was initially characterized for subgenotype by sequence comparison of the 5`untranslated region (UTR) region, as is the established process for BVDV [29, 30]. Known viruses of the BVDV1a, BVDV1b, and BVDV2a subgenotypes were included in this cross comparison (Supplemental Table 1). Based upon the 5`UTR based phylogenetic analysis with known BVDV subgenotypes, UCD-16 was closely related to the BVDV1a Singer strain, which is widely used in commercial vaccines (Fig. 2A). During viral culture and isolation on Madin-Darby Bovine Kidney (MDBK) and Bovine Turbinate (BTu) cells cytopathic effect (CPE) was observed (data not shown) indicating that UCD-16 is of the CP biotype and was not derived from a persistent infection. A phylogenetic analysis with all publicly available BVDV-1 strains was completed (Supplemental File 2), and it also demonstrated that the Singer strain (NCBI accession: MH133206.1) is the most closely related BVDV sequence. Full genome alignment of UCD-16 with Singer (MH133206.1) is 99.9% identical with a total of 12 single nucleotide polymorphisms (SNPs) across the 12.3-kb genome. Of these 12 base differences, three SNPs were silent, and nine lead to amino acid changes (Fig. 2B, C). Of the nine amino acid changes, seven impacted the polarity, as shown by the isoelectric point (D695H, E708K, D777N, P816S, E1015K, E2792K, D3140N) (Fig. 2B), and most had minimal impact on residue hydrophobicity except for P816S (Fig. 2C). In order of genomic position, the first two amino acid changes are within the E1 gene region, the next five are within the E2 gene region, and the last 2 are in the NS5A gene region.

## Discussion

Here, we report the genomes of *M. bovis, M. haemolytica*, and BVDV, which were isolated from the pneumonic lung of a diseased bovine. Recognizing the co-occurrence of bacterial and viral pathogens is important, as BRD is a multifactorial disease complex. Modeling BRD through experimental sequential and co-infection studies is often challenging, making isolation of naturally occurring co-infections particularly important [31–35]. Although the *M. haemolytica* genome presented no significant findings, further work is needed to phenotypically characterize the virulence of the bacterial isolates. The virulence of the isolated *M. bovis* and *M. haemolytica* strains acting independently remains unknown.

Our analysis found that *M. bovis* strain BC10 shares a most common recent ancestor with PG45, also known as Donetta, which is the *M. bovis* type strain [22, 23]. This shared most common recent ancestor suggests that PG45 may be representative of some circulating *M. bovis* strains. Additionally, we found the presence of virulence-associated genes carried by BC10 to be consistent with most of the sequenced strains. The close relationship of BC10 to *M. bovis* PG45 is noteworthy, as bacteria rapidly adapt to in vitro conditions and concerns have been raised about the adequacy of using laboratory-adapted reference strains when drawing conclusions about pathogenesis [36].

Indeed, it has been suggested that no one bacterial strain can truly represent the scope of its entire species [36]. In this study, *M. bovis* BC10 was identified as a novel ST, ST299. However, the results also indicated that the MLST was not representative of the core genome phylogenetic tree, which is consistent with prior work [37]. Additionally, the results suggest that *M. bovis* continues to demonstrate genomic plasticity when examining individual genes, as BC10 has a greater number of highly variable surface lipoproteins, all of which are distinct from PG45. The variable surface lipoproteins have been proposed to play a role in complex adherence-mediated regions in pathogenesis [38]. Previous reports have found extensive sequence variations in *vsp* genes, demonstrating the great potential for antigenic variation [39]. The distribution of variable surface lipoproteins has been suggested to be associated with host factors such as animal age, animal breed, and the type of operation [40]. The greater diversity and number of variable surface lipoprotein genes in BC10 may play a role in resisting host immune defense, suggesting a need to better understand how these features may contribute to disease severity during co-infection.

The CP BVDV viral isolate, UCD-16, is nearly identical to the well characterized Singer strain, which is included in many commercially available vaccines, including modified live vaccines [17, 41]. One of the pitfalls of modified live vaccines is their ability to generate a virulent form of the attenuated vaccine strain [42]. While unlikely, vaccine-derived virulent viruses have been documented, most notably illness-causing vaccine-derived polio viruses derived from the oral polio vaccine [43]. Whether the BVDV infection is a product of the BRD bacterial co-infection of a naturally occurring Singer variant or due to the point mutations between UCD-16 and the Singer vaccine strain remains to be determined. The ability of this isolate and its distinct mutations to subvert antibodies to the Singer strain and immune response is an area of active research.

In conclusion, the genomes of *M. bovis, M. haemolytica*, and BVDV, which were isolated from the lung of a diseased animal, are presented here. The acknowledgement of the co-occurrence is relevant and important, as BRD is a multifactorial disease complex. Further work should investigate any influence of the point mutations between the UCD-16 and the Singer strain on host-pathogen interactions.

## Methods

### Viral isolation

Pneumonic lung tissue was cut, and fluid from the tissue was saturated in two swabs. Fluid from the tissue swabs was transferred to tubes containing phosphate buffered saline (PBS) supplemented with Antibiotic-Antimycotic (Gibco, Waltham, MA, USA). Tissue fluid was inoculated into MDBK and BTu cells for 1 hour at 37 °C and 5% CO_2_ (Gibco MEM 11095 supplemented with 10% filtered and heat-inactivated fetal bovine serum (FBS)). Inoculum was removed and cell monolayers were washed twice with PBS before incubation for 4 days at 37 °C and 5% prior to freezing at −80 °C. Appearance or absence of CPE was observed visually by light microscopy each day during 4 day incubation. Frozen inoculated cells were thawed for a second passage. Cell supernatant was removed and inoculated again onto MDBK or BTu cells for 1 hour at 37 °C and 5% CO_2_. Complete medium was added and inoculated cells were incubated at 37 °C and 5% CO_2_ for 4 days before freezing at −80 °C. Second passage frozen plates were subsequently thawed for viral RNA extraction for sequencing.

### Bacterial isolation

Pneumonic lung tissue and lung swabs were spread on both BD Difco PPLO agar (Franklin Lakes, NJ, USA) supplemented with horse serum (Gibco), ampicillin (Sigma-Aldrich, St. Louis, MO, USA), sodium pyruvate (Sigma-Aldrich), glucose (Sigma-Aldrich), and yeast extract (FisherScientific, Pittsburgh, PA, USA) and BD Difco Columbia agar supplemented with 5% defibrinated bovine blood, vancomycin (Sigma-Aldrich), and amikacin (Sigma-Aldrich). Plates were incubated at 37 °C and 10% CO_2_. After 24 hours, two morphologies were observed on the PPLO and Columbia agar plates. The predominant morphology was identified as *M. haemolytica,* which was confirmed as serotype 1 using a rabbit serum plate agglutination assay [44] and was found to be PCR positive for Leukotoxin D. The primers for Leukotoxin D detection were 5’-gagtccttgccggtgattga-3’ and 5’- aggcctgtctgcgttgatta-3’, which post-amplification resulted in a product of 493 bp. A 50-uL PCR reaction using the Qiagen HotStartTaq Plus Mastermix kit (Qiagen, Germantown, MD, USA) was carried out with the following amplification conditions: 95 °C for 5 minutes, 35 cycles of 95° 30s, 56° 15s, and 72° 60s, and 72° 5 minutes. PCR generated amplicons were run on a 1% agarose gel in 0.5% TBE at 100 volts for 60 minutes. Subsequently, the gel was stained in ethidium bromide for visualization. The identities of *M. haemolytica* BC2 and *M. bovis* BC10 were confirmed by MALDI-TOF using a BD Bruker MALDI Biotyper Sirius One System at the USDA’s National Veterinary Services Laboratories Diagnostic Bacteriology and Pathology Laboratory (Ames, IA, USA).

### Viral RNA extraction and DNA synthesis

Viral RNA was extracted using the QIAcube with the QIAamp Viral RNA Mini QIAcube Kit (Qiagen) following the manufacturer’s instructions. BVDV detection was confirmed using the VetMAX-Gold BVDV PI Detection Kit (Applied Biosystems, Waltham, MA, USA) using the PI28 known BVDV strain for the standard curve. Genotype was determined by 5’ UTR sequencing using RT-PCR with HCV90-368 primers, as is standard practice [29, 30]. The cDNA was generated from the viral RNA using the Maxima H Minus Double-Stranded cDNA Synthesis Kit (ThermoFisher, Waltham, MA, USA) following manufacturer’s instructions. cDNA was purified using the GeneJET PCR Purification kit (ThermoFisher Scientific).

### Bacterial DNA extraction

*M. haemolytica* BC2 was grown on supplemented Columbia bovine blood agar plates, and genomic DNA was extracted using a Roche High Pure PCR Template DNA extraction kit (Roche Diagnostics, North America, Indianapolis, IN, USA). *M. bovis* BC10 was grown in 200 mL of BD Difco PPLO broth supplemented with horse serum, ampicillin, glucose, sodium pyruvate, and yeast extract. Forty milliliters (mL) of the broth was pelleted, and DNA was extracted using the QIAamp Blood Midi DNA extraction kit following the manufacturer’s instructions (Qiagen). The extracted DNA was quantified with a Qubit fluorimeter (Invitrogen, Carlsbad, CA, USA).

### Sequencing

Short-read sequencing on the viral and bacterial isolates was performed by the National Veterinary Services Laboratories’ Diagnostic Bacteriology and Pathology Laboratory. Libraries were prepared using the Illumina DNA Prep sample method for the Illumina Nextseq 2000 platform. Sequencing was completed using 2 × 150 P1 paired-end SBS (Sequencing by synthesis) chemistry per the manufacturer’s instructions (Illumina, Inc., San Diego, CA, USA). For both *M. haemolytica* and *M. bovis*, demultiplexed short reads were trimmed with BBDuk (BBTools v. 2.30.0) [45].

Long read sequencing on the bacterial genomes was performed at the USDA National Animal Disease Center (Ames, IA) on an Oxford MinION Mk1C device (Oxford Nanopore, Oxford, UK). The genomic library was prepared using the SQK-RBK114.24 rapid barcoding kit, and the library was run on a FLO-MIN114 (R10 version) flow cell. Long reads were basecalled using MinKNOW v. 24.11.8, and reads with a quality score of less than 9 were discarded.

### Genomic assembly

The UCD-16 BVDV viral genome reads were adaptor and quality trimmed with fastp v. 0.21.0 and then *de novo* assembled with SPAdes v3.15.5 employing the –metaviral option [46, 47]. The genome assembly derived from SPAdes was subsequently used as the reference in a round of reference-based assembly using VIGA to improve assembly at the termini of the linear genome [48]. Error correction algorithms showed no further base corrections were indicated (data not shown).

Long read sequences were assembled with Flye v. 2.9.4 [49]. The Flye assemblies were polished with the long reads using Medaka v. 2.0.1 [50]. Subsequently, the Medaka polished assemblies were polished with Polypolish v. 0.6.0 [51] using the short reads trimmed with BBDuk [45]. Long read assemblies were examined in Geneious Prime v. 2021.1.1 [52], and validated by mapping the long reads back to the polished assemblies. The species identity of the isolates was confirmed by using KmerFinder 3.2 [53–55] on the polished assemblies.

### Genomic investigation

On March 20, 2025, the genomic assemblies for *M. haemolytica* were downloaded from NCBI’s Genome/Assembly page. Genomes were excluded if they were identified by NCBI as metagenome-associated genomes, atypical genomes, or genomes from large multi-isolate projects. The resultant genomes (*n* = 382) with our *M. haemolytica* isolate were annotated with Prokka [56], and the core genome was obtained using Roary [57]. Genomes with duplicate core genomes were excluded from the analysis. A maximum likelihood tree was produced using RAxML with a GTRGAMMA model [58]. The tree was midpoint rooted and visualized in iTOL [59, 60]. The multilocus sequence typing was obtained using the MLST program [8, 61, 62]. A custom BLAST database was used to compare the *M. haemolytica* virulence associated genes among the genomes. The serotypes were assigned if there was 100% identity to the sequences on the ivsmlst BIGSdb website (https://ivsmlst.sund.ku.dk/) [63]. Antimicrobial resistance genes were queried using ABRicate with the ResFinder database [64, 65]. The queried sequences and genomic assembly workflow are available at http://www.github.com/nielsend/BRDC_case.

On March 3, 2025, the genomic assemblies for *M. bovis* were downloaded from NCBI’s Genome/Assembly page. Genomes were excluded if they were identified by NCBI as metagenome-associated genomes, atypical genomes, or genomes from large multi-isolate projects. The resultant genomes (*n* = 184) with our *M. bovis* isolate were annotated with Prokka [56], and the core genome was obtained using Roary [57]. Genomes with a duplicate core genome were dropped. A maximum likelihood tree was produced using RAxML with a GTRGAMMA model [58]. The tree was midpoint rooted and visualized in iTOL [59, 60]. The multilocus sequence typing was analyzed using the MLST program [8, 61, 62]. A custom BLAST database was used to compare previously identified *M. bovis* adhesins or virulence-associated genes among the genomes [26]. Variable surface lipoproteins were identified following annotation with PGAP (build 7555) [66]. Antimicrobial resistance genes were queried using ABRicate with the ResFinder database [64, 65]. A comparative analysis of the genomic architecture for *M. bovis* strains (BC10 with accessions NC_014760.1 and CP002058.1) and *M. agalactiae* PG2 (accession NC_009497.1) were performed using Mauve genome alignment software v. 1.1.3 [67].

Genotypic classification of the assembled BVDV isolate was conducted via alignment of the 5` UTR sequence of the UCD-16 isolate with known BVDV1a, BVDV1b, and BVDV2a sequences (Supplemental Table 1), as is the established practice [29, 30]. On July 8, 2025, all “complete” nucleotide sequences for BVDV-1 were downloaded from the NCBI Virus database (*n* = 145). With these sequences, a phylogenetic tree was constructed using Molecular Evolutionary Genetics Analysis 12 (MEGA12) software tool’s ClustalW alignment and a maximum likelihood method (Supplemental File 2) [68]. ClustalW alignment and a maximum likelihood method were also used to generate a phylogenetic tree with branch support estimated via 1,000 bootstrap replicates and Poisson correction for the calculation of evolutionary distance for a selection of BVDV strains [68]. In addition to 5’ UTR sequencing, the complete UCD-16 BVDV isolate genome was compared against the BVDV1a Singer strain complete genome sequence.

## Electronic supplementary material

Below is the link to the electronic supplementary material.

Supplementary Material 1

Supplementary Material 2

Supplementary Material 3

Supplementary Material 4: Figure S1

Supplementary Material 5: Figure S2

## Data Availability

The sequencing reads and bacterial genomes can be found in the PRJNA1370137 and PRJNA1370143 NCBI BioProjects. BVDV UCD-16 can be found at NCBI as accession number PX619761 (https://www.ncbi.nlm.nih.gov/nuccore/PX619761.1/).

## References

[1] Taylor JD, Fulton RW, Lehenbauer TW, Step DL, Confer AW. The epidemiology of bovine respiratory disease: what is the evidence for predisposing factors? Can Vet J. 2010;51(10):1095–102. PMC2942046 21197200

[2] Cusack PM, McMeniman N, Lean IJ. The medicine and epidemiology of bovine respiratory disease in feedlots. Aust Vet J. 2003;81(8):480–87. 10.1111/j.1751-0813.2003.tb13367.x 15086084

[3] Kamel MS, Davidson JL, Verma MS. Strategies for bovine respiratory disease (BRD) diagnosis and prognosis: a comprehensive overview. Anim (Basel). 2024;14(4):627. 10.3390/ani14040627. PMC10885951 38396598

[4] Centeno-Martinez RE, Glidden N, Mohan S, Davidson JL, Fernández-Juricic E, Boerman JPSchoonmaker J, et al. Identification of bovine respiratory disease through the nasal microbiome. Anim Microbiome. 2022;4(1). 10.1186/s42523-022-00167-y. PMC8862248 35193707

[5] Johnston D, Earley B, Cormican P, Murray G, Kenny DA, Waters SM, et al. Illumina MiSeq 16S amplicon sequence analysis of bovine respiratory disease associated bacteria in lung and mediastinal lymph node tissue. BMC Vet Res. 2017;13(1):118. 10.1186/s12917-017-1035-2. PMC5414144 28464950

[6] Holman DB, McAllister TA, Topp E, Wright ADG, Alexander TW. The nasopharyngeal microbiota of feedlot cattle that develop bovine respiratory disease. Vet Microbiol. 2015;180(1–2):90–95. 10.1016/j.vetmic.2015.07.031. 26249828

[7] Maunsell FP, Woolums AR, Francoz D, Rosenbusch RF, Step DL, Wilson DJ, et al. Mycoplasma bovis infections in cattle. J Vet Intern Med. 2011;25(4):772–83. 10.1111/j.1939-1676.2011.0750.x. 21745245

[8] Register KB, Lysnyansky I, Jelinski MD, Boatwright WD, Waldner M, Bayles DO, et al. Comparison of two multilocus sequence typing schemes for Mycoplasma bovis and revision of the PubMLST reference method. J Clin Microbiol. 2020;58(6). 10.1128/JCM.00283-20. PMC7269390 32295891

[9] Rosales RS, Churchward CP, Schnee C, Sachse K, Lysnyansky I, Catania S, et al. Global multilocus sequence typing analysis of Mycoplasma bovis isolates reveals two main population clusters. J Clin Microbiol. 2015;53(3):789–94. 10.1128/JCM.01910-14. PMC4390661 25540400

[10] Register KB, Thole L, Rosenbush RF, Minion FC. Multilocus sequence typing of Mycoplasma bovis reveals host-specific genotypes in cattle versus bison. Vet Microbiol. 2015;175(1):92–98. 10.1016/j.vetmic.2014.11.002. 25433454

[11] Grooms DL. Reproductive consequences of infection with bovine viral diarrhea virus. Vet Clin N Am Food Anim Pract. 2004;20(1):5–19. 10.1016/j.cvfa.2003.11.006. 15062471

[12] Riley JM, Peel DS, Raper KC, Hurt C. INVITED REVIEW: economic consequences of beef cow-calf disease mismanagement: bovine viral diarrhea virus. Appl Anim Sci. 2019;35(6):606–14. 10.15232/aas.2019-01861.

[13] Simmonds P, Becher P, Bukh J, Gould EA, Meyers G, Monath T, et al. ICTV virus taxonomy Profile: flaviviridae. J Gener Virol. 2017;98(1):2–3. 10.1099/jgv.0.000672. PMC5370391 28218572

[14] Yesilbag K, Alpay G, Becher P. Variability and Global distribution of subgenotypes of bovine viral diarrhea virus. Viruses-Basel. 2017;9(6):128. 10.3390/v9060128. PMC5490805 28587150

[15] Coggins L, Wagner WC, Baker JA, Gillespie JH, Phillips WV, Robson DS, et al. Attenuation of virus diarrhea virus (strain Oregon C24v) for vaccine purposes. Cornell Vet. 1961;51(4):539±. 13880178

[16] van Oirschot J, Bruschke CJM, van Rijn PA. Vaccination of cattle against bovine viral diarrhoea. Vet Microbiol. 1999;64(2–3):169–83. 10.1016/S0378-1135(98)00268-5. 10028171

[17] Fulton RW, Whitley EM, Johnson BJ, Ridpath JF, Kapil S, Burge LJ, et al. Prevalence of bovine viral diarrhea virus (BVDV) in persistently infected cattle and BVDV subtypes in affected cattle in beef herds in south central United States. Can J Vet Res. 2009;73(4):283–91. PMC2757709 20046630

[18] Baker JC. The clinical manifestations of bovine viral diarrhea infection. Vet Clin N Am Food Anim Pract. 1995;11(3):425–45. 10.1016/S0749-0720(15)30460-6. 8581856

[19] Peterhans E, Bachofen C, Stalder H, Schweizer M. Cytopathic bovine viral diarrhea viruses (BVDV): emerging pestiviruses doomed to extinction. Vet Res. 2010;41(6):44. 10.1051/vetres/2010016. PMC2850149 20197026

[20] Bell RL, Turkington HL, Cosby SL. The bacterial and viral agents of BRDC: immune evasion and vaccine developments. Vaccines-Basel. 2021;9(4):337. 10.3390/vaccines9040337. PMC8066859 33916119

[21] Gaudino M, Nagamine B, Ducatez MF, Meyer G. Understanding the mechanisms of viral and bacterial coinfections in bovine respiratory disease: a comprehensive literature review of experimental evidence. Vet Res. 2022;53(1). 10.1186/s13567-022-01086-1. PMC9449274 36068558

[22] Hale HH, Helmboldt CF, Plastridge WN, Stula EF. Bovine mastitis caused by a Mycoplasma species. Cornell Vet. 1962;52:582–91. 13952069

[23] Edward DGF, Freundt E. Type strains of species of the order mycoplasmatales, including designation of neotypes for Mycoplasma mycoides subsp. mycoides, Mycoplasma agalactiae subsp. agalactiae, and Mycoplasma arthritidis. Int J Syst Bacteriol. 1973;23(1):55–61. 10.1099/00207713-23-1-55.

[24] Wise KS, Calcutt MJ, Foecking MF, Roske K, Madupu R, Methe BA. Complete genome sequence of Mycoplasma bovis type strain PG45 (ATCC 25523). (ATCC 25523). Infect Immun. 2011;79(2):982–83. 10.1128/IAI.00726-10. PMC3028869 21134966

[25] Qi J, Guo A, Cui P, Chen Y, Mustafa R, Ba X, et al. Comparative geno-plasticity analysis of Mycoplasma bovis HB0801 (Chinese isolate). PLoS One. 2012;7(5):e38239. 10.1371/journal.pone.0038239. PMC3365025 22693604

[26] Xu QY, Pan Q, Wu Q, Xin J. Mycoplasma bovis adhesins and their target proteins. Front Immunol. 2022;13. 10.3389/fimmu.2022.1016641 PMC9630594 36341375

[27] Adamu JY, Wawegama NK, Browning GF, Markham PF. Membrane proteins of Mycoplasma bovis and their role in pathogenesis. Res Vet Sci. 2013;95(2):321–25. 10.1016/j.rvsc.2013.05.016. 23810376

[28] Burki S, Frey J, Pilo P. Virulence, persistence and dissemination of Mycoplasma bovis. Vet Microbiol. 2015;179(1–2):15–22. 10.1016/j.vetmic.2015.02.024 25824130

[29] Ridpath JF, Bolin SR, Dubovi EJ. Segregation of bovine viral diarrhea virus into genotypes. Virology. 1994;205(1):66–74. 10.1006/viro.1994.1620. 7975238

[30] Bauermann FV, Flores EF, Ridpath JF. Antigenic relationships between bovine viral diarrhea virus 1 and 2 and HoBi virus: possible impacts on diagnosis and control. J Vet Diagn Invest. 2012;24(2):253–61. 10.1177/1040638711435144. 22379042

[31] Baldwin DE, Marshall RG, Wessman GE. Experimental infection of calves with myxovirus parainfluenza-3 and Pasteurella haemolytica. Am J Vet Res. 1967;28(127):1773–82. 4294767

[32] Gånheim C, Hultén C, Carlsson U, Kindahl H, Niskanen R, Waller KP. The acute Phase response in calves experimentally infected with bovine viral diarrhoea virus and/or Mannheimia haemolytica. J Vet Med, Ser B. 2003;50(4):183–90. 10.1046/j.1439-0450.2003.00658.x. 12916692

[33] Gershwin LJ, Berghaus LJ, Arnold K, Anderson ML, Corbeil LB. Immune mechanisms of pathogenetic synergy in concurrent bovine pulmonary infection with Haemophilus somnus and bovine respiratory syncytial virus. Vet Immunol Immunopathol. 2005;107(1–2):119–30. 10.1016/j.vetimm.2005.04.004 15979157

[34] Sudaryatma PE, Mekata H, Kubo M, Subangkit M, Goto Y, Okabayashi T. Co-infection of epithelial cells established from the upper and lower bovine respiratory tract with bovine respiratory syncytial virus and bacteria. Vet Microbiol. 2019;235:80–85. 10.1016/j.vetmic.2019.06.010. 31282382

[35] Lin C, Agnes JT, Behrens N, Shao M, Tagawa Y, Gershwin LJ, et al. Histophilus somni stimulates Expression of Antiviral proteins and Inhibits BRSV Replication in bovine respiratory epithelial cells. PLoS One. 2016;11(2):e0148551. 10.1371/journal.pone.0148551. PMC4747540 26859677

[36] Fux CA, Shirtliff M, Stoodley P, Costerton JW. Can laboratory reference strains mirror ‘real-world’ pathogenesis? Trends Microbiol. 2005;13(2):58–63. 10.1016/j.tim.2004.11.001 15680764

[37] Menghwar H, Prysliak T, Perez-Casal J. Phylogeny of Mycoplasma bovis isolates from cattle and bison based on multi locus sequence typing and multiple-locus variable-number tandem repeats. Vet Microbiol. 2021;258:109124. 10.1016/j.vetmic.2021.109124. 34058524

[38] Sachse K, Helbig JH, Lysnyansky I, Grajetzki C, Muller W, Jacobs E, et al. Epitope mapping of immunogenic and adhesive structures in repetitive domains of Mycoplasma bovis variable surface lipoproteins. Infect Immun. 2000;68(2):680–87. 10.1128/IAI.68.2.680-687.2000. PMC97192 10639433

[39] Nussbaum S, Lysnyansky I, Sachse K, Levisohn S, Yogev D. Extended repertoire of genes encoding variable surface lipoproteins in Mycoplasma bovis strains. Infect Immun. 2002;70(4):2220–25. 10.1128/IAI.70.4.2220-2225.2002. PMC127842 11895991

[40] Dudley EP, Scott MA, Kittana H, Thompson AC, Valeris-Chacin R. The pathogenomics of the respiratory Mycoplasma bovis strains circulating in cattle around the Texas Panhandle, USA. Pathogens. 2025;14(2):167. 10.3390/pathogens14020167. PMC11858780 40005542

[41] Fulton RW, Cook BJ, Payton ME, Burge LJ, Step DL. Immune response to bovine viral diarrhea virus (BVDV) vaccines detecting antibodies to BVDV subtypes 1a, 1b, 2a, and 2c. Vaccine. 2020;38(24):4032–37. 10.1016/j.vaccine.2020.03.058 32336600

[42] d’Offay JM, Fulton RW, Fishbein M, Eberle R, Dubovi EJ. Isolation of a naturally occurring vaccine/wild-type recombinant bovine herpesvirus type 1 (BoHV-1) from an aborted bovine fetus. Vaccine. 2019;37(32):4518–24. 10.1016/j.vaccine.2019.06.059. 31266667

[43] Namageyo-Funa A, Greene SA, Henderson E, Traore MA, Shaukat S, Bigouette JP, et al. Update on vaccine-derived poliovirus outbreaks — worldwide, January 2023–June 2024. MMWR Morb Mortal Wkly Rep. 2024;73(41):909–16. 10.15585/mmwr.mm7341a1. PMC11486351 39418214

[44] Frank GH, Wessman GE. Rapid plate agglutination procedure for serotyping Pasteurella haemolytica. J Clin Microbiol. 1978;7(2):142–45. 10.1128/jcm.7.2.142-145.1978. PMC274882 632345

[45] Bushnell B, Rood J, Singer E. Bbmerge – accurate paired shotgun read merging via overlap. PLoS One. 2017;12(10):e0185056. 10.1371/journal.pone.0185056. PMC5657622 29073143

[46] Bankevich A, Nurk S, Antipov D, Gurevich AA, Dvorkin M, Kulikov AS, et al. Spades: a New genome assembly algorithm and its applications to single-cell sequencing. J Comput Biol. 2012;19(5):455–77. 10.1089/cmb.2012.0021. PMC3342519 22506599

[47] Chen SF, Zhou YQ, Chen YR, Gu J. Fastp: an ultra-fast all-in-one FASTQ preprocessor. Bioinformatics. 2018;34(17):i884–90. 10.1093/bioinformatics/bty560. PMC6129281 30423086

[48] Fu P, Wu YF, Zhang ZY, Qiu Y, Wang YR, Peng YS. VIGA: a one-stop tool for eukaryotic virus identification and genome assembly from next-generation-sequencing data. Brief Bioinform. 2024;25(1). 10.1093/bib/bbad444 PMC10753531 38048079

[49] Kolmogorov M, Yuan J, Lin Y, Pevzner PA. Assembly of long, error-prone reads using repeat graphs. Nat Biotechnol. 2019;37(5):540–46. 10.1038/s41587-019-0072-8 30936562

[50] Lee JY, Kong M, Oh J, Lim J, Chung SH, Kim JM, et al. Comparative evaluation of Nanopore polishing tools for microbial genome assembly and polishing strategies for downstream analysis. Sci Rep. 2021;11(1):20740. 10.1038/s41598-021-00178-w. PMC8528807 34671046

[51] Wick RR, Holt KE. Polypolish: short-read polishing of long-read bacterial genome assemblies. PLoS Comput Biol. 2022;18(1):e1009802. 10.1371/journal.pcbi.1009802 PMC8812927 35073327

[52] Kearse M, Moir R, Wilson A, Stones-Havas S, Cheung M, Sturrock S, et al. Geneious basic: an integrated and extendable desktop software platform for the organization and analysis of sequence data. Bioinformatics. 2012;28(12):1647–49. 10.1093/bioinformatics/bts199. PMC3371832 22543367

[53] Hasman H, Saputra D, Sicheritz-Ponten T, Lund O, Svendsen CA, Frimodt-Moller N, et al. Rapid whole-genome sequencing for detection and characterization of microorganisms directly from clinical samples. J Clin Microbiol. 2014;52(1):139–46. 10.1128/JCM.02452-13. PMC3911411 24172157

[54] Clausen P, Aarestrup FM, Lund O. Rapid and precise alignment of raw reads against redundant databases with KMA. BMC Bioinf. 2018;19(1):307. 10.1186/s12859-018-2336-6. PMC6116485 30157759

[55] Larsen MV, Cosentino S, Lukjancenko O, Saputra D, Rasmussen S, Hasman H, et al. Benchmarking of methods for genomic taxonomy. J Clin Microbiol. 2014;52(5):1529–39. 10.1128/JCM.02981-13. PMC3993634 24574292

[56] Seemann T. Prokka: rapid prokaryotic genome annotation. Bioinformatics. 2014;30(14):2068–69. 10.1093/bioinformatics/btu153. 24642063

[57] Page AJ, Cummins CA, Hunt M, Wong VK, Reuter S, Holden MTG, et al. Roary: rapid large-scale prokaryote pan genome analysis. Bioinformatics. 2015;31(22):3691–93. 10.1093/bioinformatics/btv421. PMC4817141 26198102

[58] Stamatakis A. RAxML version 8: a tool for phylogenetic analysis and post-analysis of large phylogenies. Bioinformatics. 2014;30(9):1312–13. 10.1093/bioinformatics/btu033 PMC3998144 24451623

[59] Letunic I, Bork P. Interactive tree of Life (iTOL): an online tool for phylogenetic tree display and annotation. Bioinformatics. 2007;23(1):127–28. 10.1093/bioinformatics/btl529 17050570

[60] Letunic I, Bork P. Interactive tree of Life (iTOL) v6: recent updates to the phylogenetic tree display and annotation tool. Nucleic Acids Res. 2024;52(W1):W78–82. 10.1093/nar/gkae268. PMC11223838 38613393

[61] Seemann T mlst.

[62] Jolley KA, Maiden MC. Bigsdb: scalable analysis of bacterial genome variation at the population level. BMC Bioinf. 2010;11:595. 10.1186/1471-2105-11-595 PMC3004885 21143983

[63] Christensen H, Bisgaard M, Menke T, Liman M, Timsit E, Foster G, et al. Prediction of Mannheimia haemolytica serotypes based on whole genomic sequences. Vet Microbiol. 2021;262:109232. 10.1016/j.vetmic.2021.109232. 34509701

[64] Bortolaia V, Kaas RS, Ruppe E, Roberts MC, Schwarz S, Cattoir V, et al. ResFinder 4.0 for predictions of phenotypes from genotypes. The J Antimicrob Chemother. 2020;75(12):3491–500. 10.1093/jac/dkaa345. PMC7662176 32780112

[65] Seemann T. Abricate: Github; [Available from: https://github.com/tseemann/abricate.

[66] Li W, O’Neill KR, Haft DH, DiCuccio M, Chetvernin V, Badretdin A, et al. RefSeq: expanding the prokaryotic genome annotation pipeline reach with protein family model curation. Nucleic Acids Res. 2021;49(D1):D1020–28. 10.1093/nar/gkaa1105. PMC7779008 33270901

[67] Darling ACE, Mau B, Blattner FR, Perna NT. Mauve: multiple alignment of conserved genomic sequence with rearrangements. Genome Res. 2004;14(7):1394–403. 10.1101/gr.2289704. PMC442156 15231754

[68] Kumar S, Stecher G, Suleski M, Sanderford M, Sharma S, Tamura K. MEGA12: molecular evolutionary genetic analysis version 12 for adaptive and green computing. Mol Biol Evol. 2024;41(12). 10.1093/molbev/msae263 PMC11683415 39708372

