# Supplementary material for: Genomic characterization of Mycoplasma bovis, Mannheimia haemolytica, and bovine viral diarrhea virus 1 from a bovine respiratory disease complex case
Source: BMC Genom Data. 2026 Jul 11;27:84. doi: 10.1186/s12863-026-01456-0 (PMC13644054; doi:10.1186/s12863-026-01456-0)
Supplement: Supplementary file 1 [file 12863_2026_1456_MOESM3_ESM.docx]

| **Supplemental Table 1. BVDV GenBank Accession Numbers** | | |
| --- | --- | --- |
| **Isolate Name** | **BVDV Sub-genotype** | **GenBank Accession #** |
| *Singer* | *1A* | *MH133206* |
| *92-27854* | *1A* | *PV626403* |
| *YandaSpl* | *1A* | *MW732739* |
| *BAOEC1190* | *1A* | *PV626381* |
| *PI407* | *1A* | *MW732738* |
| *GL760* | *1A* | *PV626380* |
| *C24V* | *1A* | *AF091605* |
| *NADL* | *1A* | *AJ133738* |
| *Trangie* | *1C* | *AF069769* |
| *Bega* | *1C* | *AF052303* |
| *TGAN* | *1B* | *PV626348* |
| *NY-1* | *1B* | *L32879* |
| *1373* | *2A* | *AF145967* |
| *890* | *2A* | *U18059* |
| UCD-16 | 1A | PX619761 |
| *GenBank Verified Sequences for Comparative Analysis Italicized* | | |
